# Up to 30 years of clinical performance of double-crown-retained removable dental prostheses with spark-eroded friction pins placed in a general dental practice

**DOI:** 10.1007/s00784-026-06971-z

**Published:** 2026-06-11

**Authors:** Karina Zierden, Seline Werdecker, Bernd Wöstmann, Peter Rehmann

**Affiliations:** 1 Private Practice, Hessen, Germany; 2 Department of Prosthodontics, School of Dental Medicine, Justus-Liebig-University, Schlangenzahl 14, Giessen, 35392 Germany

**Keywords:** Removable dental prosthesis, Double crowns, Friction pins, Survival, Risk factors

## Abstract

**Purpose:**

The purpose of this retrospective cohort study was to investigate the clinical performance and longevity of double-crown-retained removable-dental-prostheses (DCR-RDP) with spark-eroded friction pins inserted in a general dental practice and followed up over a period of 30 years.

**Materials and methods:**

This retrospective cohort study observed the influence of various factors on the longevity of the DCR-RDPs with spark-eroded friction pins and the abutment teeth using Kaplan-Meier estimation (log-rank test, *p* < .05) estimation and Cox regression (α = 0.05).

**Results:**

During a mean follow-up of 14.07 ± 7.89 years (max 31.66 years), 196 prostheses and 563 abutments were observed. 30.61% (*n* = 60) of the prostheses and 39.43% (*n* = 222) of the abutments failed. The mean survival time for the prostheses was 22.66 ± 0.86 years (95% CI:20.97 to 24.24 years), and for the abutments, 20.29 ± 0.51 years (95% CI: 19.29 to 21.29 years). The following factors had a significant influence on the performance of either the prostheses or the abutment teeth: Kennedy class, number of replaced teeth, opposing dentition, number of abutments, crown-root ratio, probing depth, location, and vitality (*p*<.05).

**Conclusions:**

The highest survival rates were shown by Kennedy Class III DCR-RDPs and abutment teeths, while the lowest were seen with prostheses with 11 or more replaced teeth. The number of abutment teeth, pocket depth, vitality, and crown/root length ratio should also be considered in terms of the abutment teeth’s longevity.

## Introduction

The double-crown retained removable partial prosthesis (DCR-RPD) is a widely used treatment method, especially in cases of reduced residual dentition. Multiple studies report good clinical performance and longevity of this type of denture [1–11]. This also applies to the longevity of the abutment teeth when treated with DCR-RPDs [3–5, 12–14].

It is a rigidly anchored partial denture based on the principle of the double-crown system. The primary crown is attached to the abutment tooth or abutment implant, while the secondary crown is part of the prosthetic framework and therefore part of the removable prosthesis. The main advantage of double crowns is that they provide rigid physical support for the abutment teeth. This physical support and the physiological axial load on the abutment teeth counteract bone atrophy of the edentulous jaw. Due to their removable design, DCR-RPDs enable optimal hygiene of the abutment teeth and prosthesis, making them particularly suitable for older people with declining motor skills [15]. However, telescopic dentures are highly advanced dental prostheses, as reflected in their manufacturing costs. These dentures also require intensive aftercare. The most common aftercare procedures are re-cementing of primary crowns, veneer repairs, and relining [9, 11, 15–17]. There are several subtypes of double crowns that differ in the materials used, preparation and retention forms, and additional retention elements. Common materials used for double crowns include precious and non-precious alloys as well as zirconia [1–3, 18].

In the present study, double-crown retained removable dental prostheses with spark-eroded friction pins (DCR-RPDs-SEFPs), who were placed in a general dental office, were observed. The unique feature of this system is that the primary and secondary crowns are made of the same material and have an additional retention element. The secondary crown is part of the framework, which eliminates the need for a tertiary structure. The alloy is a non-precious metal alloy (cobalt-chromium-molybdenum alloy), which is characterized by high biocompatibility and low allergenic potential [1–3]. The retention force of telescopic prostheses usually decreases over time, especially for non-precious metal double crowns. Therefore, it is sometimes necessary to fabricate a new prosthesis in certain cases. However, this risk does not apply to DCR-RPDS-SEFPs, since the additional retention elements can be easily reactivated.

Occasionally, this type of double crown has only been the subject of a few studies [1–3, 12, 13]. Furthermore, all of these studies were conducted in universities. The only exception was the study by Klotz et al., which took place in a dental practice [16]. Moreover, only two comparable studies with an equal observation period were found [4, 5].

## Objectives of the study

This study aimed to evaluate the clinical performance and longevity of DCR-RPDs-SEFPs in daily practice over a period of up to 30 years. This should help to identify and prevent late complications affecting the prostheses and abutment teeth survival, which are rarely observed yet. The study also aimed to identify the factors that significantly influence the longevity of the prostheses and their abutment teeth in order to draw important conclusions for the treatment and aftercare of patients with DCR-RPDs-SEFPs.

Therefore, the following null hypothesis tested was that the variables had no effect on the survival of the prostheses or the abutments.

## Materials and methods

### Study design

This retrospective longitudinal study observed the data of 196 patients who received DCR-RDPs-SEFPs at a general dental office in Germany. All prostheses are manufactured and finally placed between 1990 and 1998. During this period, all of the prostheses examined were fabricated and placed by a single dentist. The overall observation period was from 1990 up to 2023. Continuous and seamless data collection was carried out within the independent dental office.

The study protocol was approved by the Ethics Committee of the Medical Faculty of the Justus-Liebig-University Giessen, Germany (Reg. Nr. 164/11).

The following study criteria were defined to include and exclude the patient cohort: only DCR-RDPs with spark-eroded friction pins that are attached to natural teeth were included in the present study. Therefore, DCR-RDPs with different types of double crowns (e.g. parallel-sided telescopic crowns or conical crowns) or different attachments (e.g. clasps) were excluded. Implant-only or implant-tooth-supported dentures were also excluded. Patients who did not attend at least one recall appointment or receive treatment after denture placement were also excluded.

**Fig. 1:**
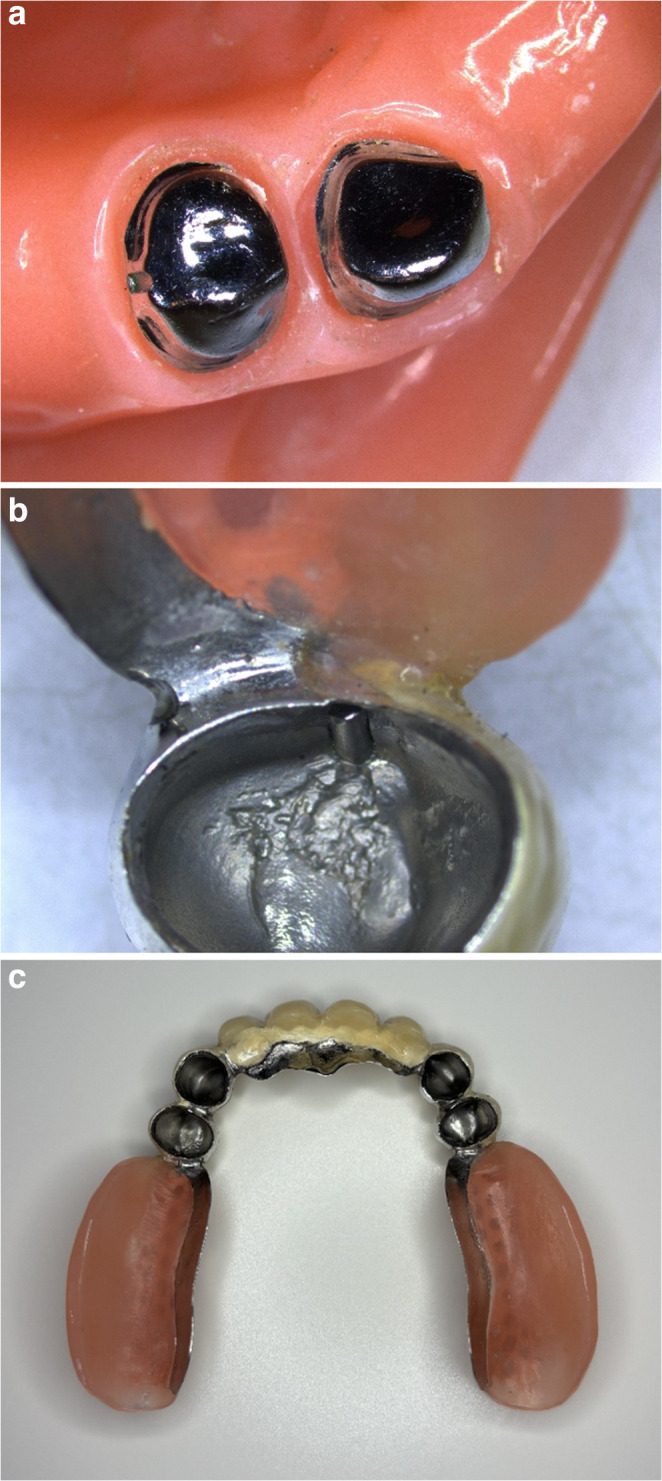
DCR-RPDs with spark-erdoded friction pins: **a**) Primary crown; **b**) Secondary crown; **c**) Complete Denture

As all DCR-RDPs-SEFPs were manufactured in the same dental laboratory, it can be assumed that the restorations are standardized and of comparable quality. Also, all prostheses were inserted by the same dentist. In cases where periodontal, conservative, or surgical pre-treatment was necessary, or where a functional diagnosis had to be performed, the appropriate treatment was carried out beforehand. Almost all of the examined dentures were attached to the remaining natural teeth. In a few cases, however, there were residual teeth that were not included in the restauration, particularly among the front teeth. The treatment procedure was as follows: The abutment teeth were prepared for taking the primary crowns by means of a chamfer preparation, and the impression was taken using a C-silicone. All dentures were made with a metal framework. The denture base and double crowns were manufactured from a non-precious metal alloy using the one-piece casting technique. Spark-eroded friction pins were incorporated into the secondary crowns (Fig. 1A-C). The friction pins press against the primary crown or a defined guide surface with controlled tension ensuring that the prosthesis holds onto the primary telescopes with a defined amount of friction. The primary crowns were cemented in place using phosphate cement. After placement of the DCR-RDPs-SEFPs, all patients were offered to participate in a regular 6-month follow-up program. The performed aftercare measures for each prosthesis were recorded after an adjustment period of three months. The study does not take into account the removal of pressure spots, as these are considered to be immediate adaptation measures.

### Statistical analysis

Kaplan-Meier analysis with 95% confidence intervals (CI) was used to estimate the probability of survival for the dentures and the abutment teeth. This allowed the 5-, 10-, 15-, 20-, 25-, and 30-year survival rates and survival times to be determined. In this study, only the log-rank test (*p*<.05) was used to assess the significance due to the long observation period, as a constant risk is assumed and the log-rank test weights events that occurred earlier in the same way as events that occurred later.

In order to determine the survival times, the date of incorporation of the prosthesis was defined as the starting point and the date of the last visit or the date of loss of function of the prosthesis or loss of the abutment tooth as the end point of the observation period.

The Cox model was used to investigate the simultaneous influence of different variables on the probability of survival (α = 0.05). The risk of loss per time was indicated by a hazard ratio.

### Variables

As possible factors influencing prosthesis and abutment survival, the following variables were defined and tested: gender (male or female or diverse); localization (mandible or maxilla), opposing dentition (group 1: natural teeth or fixed dental prosthesis, group 2: double-crown-retained prosthesis; group 3: full dentures; group 4: implant supported removable-dental-prostheses); Kennedy class I (bilateral short arches) or class II (unilateral shortened arch) or class III (lateral gap) classification; number of abutment teeth (group 1: 1–2 abutments, group 2: 3–4 abutments; group 3: 5–6 abutments, group 4: 7 and more abutments); number of replaced teeth (group 1: up to six replaced teeth, group 2: 7 to 10 replaced teeth, group 3: 11 and more replaced teeth; classification based on statistical analysis). In addition, the following factors have also been tested on the abutment teeth: probing depth (≥ 3 mm or 4 to 5 mm or ≤ 6 mm); crown-root length ratio (> 1.8:1 or ≤ 1.8:1; 1.8 shows the arithmetic mean) and vitality (vital tooth or root canal-treated tooth or root canal-treated tooth with core buildup).

## Results

### Descriptive data

According to the study criteria, 109 (55.61%) women and 87 (44.39%) men, with a total of 196 prostheses, 97 in the maxilla and 99 in the mandible (variable: location), were included in the study. The mean age of the participants at the time of denture delivery was 61.73 ± 9.57 years (range 36 to 80 years). Before treatment started, 121 patients had a Kennedy Class I (bilateral shortened dental arch), 50 patients had a Kennedy Class II (unilateral shortened dental arch), and 25 patients had a Kennedy Class III (multiple gaps per dental arch) distribution of abutment teeth (variable: Kennedy Class). 74 (37.76%) patients had 1 or 2 abutment teeth, 108 (55.10%) patients had 3 to 4 abutment teeth, 11 (5.61%) patients had 5 to 6 abutments, and 3 (1.53%) patients had 7 or more abutments (variable: number of abutment teeth). 37 (18.8%) prostheses replaced up to six teeth, 79 (40.3%) prostheses replaced 7 to 10 teeth, and 80 prostheses replaced 11 or more teeth (variable replaced teeth per denture). In the opposing dentition, 47 patients had natural teeth or fixed dental prostheses, 102 had DCR-RPDs, 36 had full dentures, and 11 patients had implant-supported removable dental prostheses (variable: opposing dentition).

Overall, 563 abutment teeth (273 maxillary and 290 mandibular) were observed. 127 abutment teeth had a probing depth ≤ 3 mm, 328 abutments had 4 to 5 mm, and 108 had a probing depth ≥ 6 mm. The crown-to-root length ratio was > 1.8:1 for 162 abutment teeth and ≤ 1.8:1 for 401 abutments. 516 abutment teeth were vital, 38 were root canal treated, and nine were root canal treated with a core build-up.

Table 1 displays the number of DCR-RPDs-SEFPs and the number of abutment teeth categorized by years under observation.

**Table 1:** Number of DCR-RPDs with spark-eroded friction pins and number of abutment teeth under observation categorized by years

Years	DCR-RPDs (*n* = 196)	Abutment teeth (*n* = 563)
5	175	470
10	131	331
15	87	222
20	48	110
25	26	62
30	8	19

## Main results

### DCR-RDPs with spark-eroded friction pins

Within the mean follow-up of 14.07 ± 7.89 years (max 31.66 years), 196 DCR-RDPs-SEFPs were observed, of which 30.61% (*n* = 60) failed. Mean survival time for the DCR-RDPs-SEFPs was 22.66 ± 0.86 years (95%CI: 20.97 to 24.34 years). The 5-, 10-, 15-, 20-, 25-, and 30-year survival times of all DCR-RPDs-SEFPs are displayed in Table 2. All failed DCR-RDPs-SEFPs were replaced with full dentures.

**Table 2:** Survival probabilities of all DCR-RPDs with spark-eroded friction pins (n=196) and all abutment teeth (n=563)

Years	DCR-RPDs (*n* = 196)	Abutment teeth (*n* = 563)
5	97.9%	94.2%
10	86.0%	81.6%
15	75.3%	67.7%
20	58.9%	48.8%
25	51.2%	38.8%
30	42.0%	31.4%

The survival time of the DCR-RDPs-SEFPs was significantly influenced by the Kennedy class variable (*p*<.05). The mean survival time of DCR-RDPs-SEFPs was 21.64 ± 1.12 years (95% CL: 19.43 to 23.84 years) for Kennedy Class I, 20.38 ± 1.52 years (95% CL: 17.40 to 23.37 years) for Kennedy Class II, and 28.76 ± 1.53 years (95% CL: 20.88 to 24.35 years) for Kennedy Class III. DCR-RDP-SEFPs survival rates related to the Kennedy class are shown in Table 3. The 90% probability of survival was not reached after 8.81 years for the Kennedy Class I prostheses, after 7.38 years for the Kennedy Class II prostheses, and after 24.36 years for the Kennedy Class III prostheses. The probability of survival fell below 50% after 21.71 years for Kennedy class I and 18.40 years for Kennedy class II. For Kennedy Class III prostheses, the probability of survival did not fall below 50%.

**Table 3:** Survival probabilities of all DCR-RPDs (n=196) and all abutment teeth (n=563) depending on Kennedy class distribution

Years	Kennedy Class I		Kennedy Class II		Kennedy Class III	
	DCR-RPDs (*n* = 121)	Abutments (*n* = 347)	DCR-RPDs (*n* = 50)	Abutments (*n* = 140)	DCR-RPDs (*n* = 25)	Abutments (*n* = 76)
5	98.3%	95.1%	93.6%	87.7%	100%	98.6%
10	85.5%	82.2%	82.0%	74.0%	90.9%	88.3%
15	71.2%	64.7%	68.4%	60.3%	90.9%	84.9%
20	55.5%	47.8%	42.1%	36.8%	90.9%	69.4%
25	46.9%	36.3%	42.1%	30.2%	80.8%	59.2%
30	30.3%	24.8%	/	/	80.8%	56.4%

**Fig. 2:**
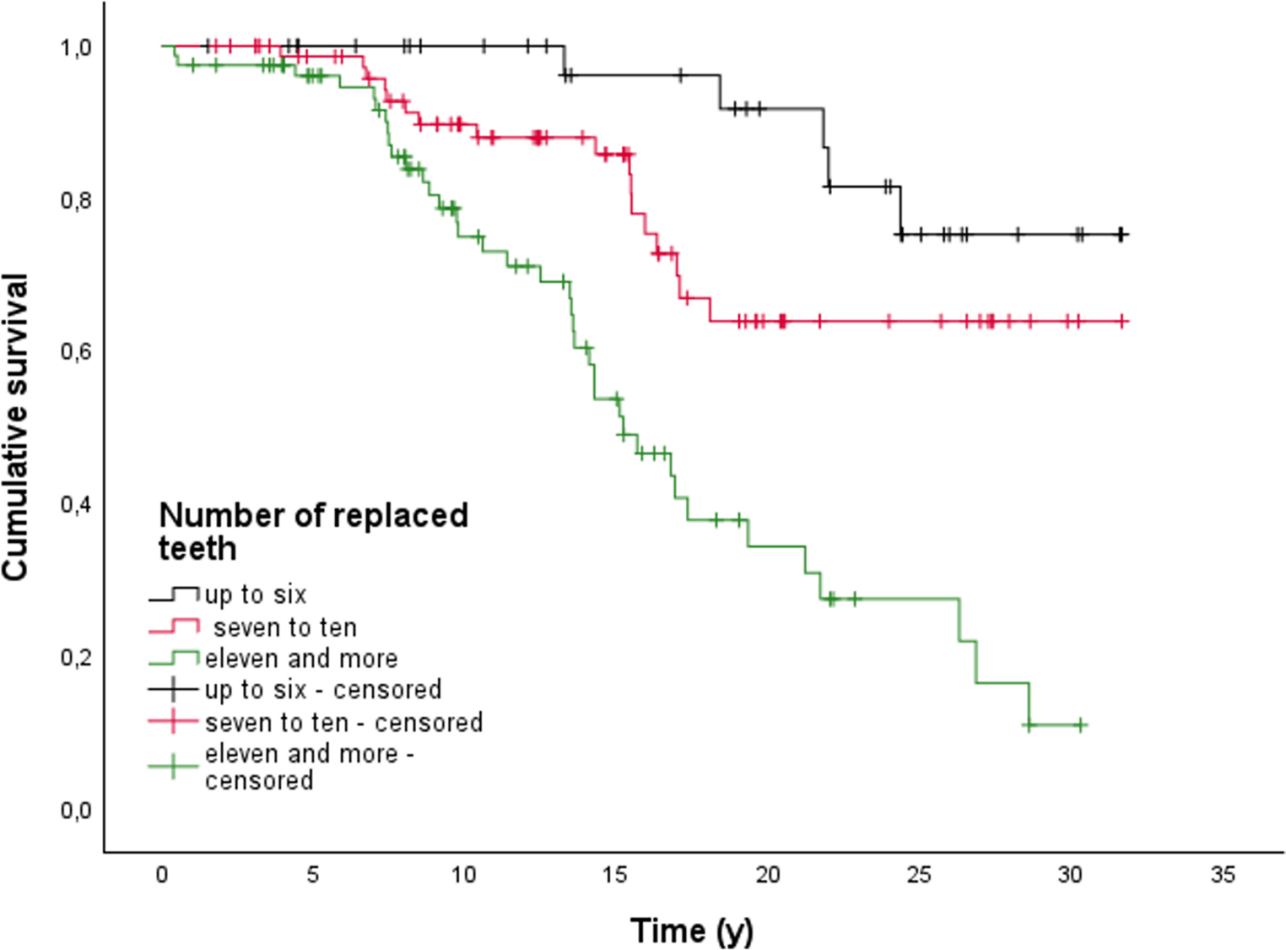
Outcome probability of all DCR-RPDs dependent on number of replaced teeth (target event: renewal, n=196; Kaplan-Meier)

The number of teeth replaced per prosthesis had a highly significant effect on the survival time of the dentures (*p* <.001). The mean survival time for DCR-RDPs-SEFPs in group 1 was 28.92 ± 1.1 years (95% CL: 26.76 to 31.07 years), for DCR-RDPs-SEFPs in group 2 the mean survival time was 25.04 ± 1.33 years (95% CL: 22.43 to 27.64 years), and for DCR-RDPs-SEFPs in group 3 it was 17.14 ± 1.18 years (95% CL: 14.83 to 19.45 years). The survival rates of the DCR-RDPs-SEFPs related to the number of teeth replaced per denture are shown in Fig. 2.

Like the univariate comparison, Kennedy class and number of teeth replaced also had a significant effect on time to loss of function in the Cox model. DCR-RDPs-SEFPs in Kennedy Class II jaws had a threefold increased risk of loss of function in comparison to Kennedy Class I. If 7 to 10 teeth (group 2) or 11 or more teeth (group 3) were replaced per prosthesis, the risk of loss of function was 4 times and 18 times higher in comparison to group 1 (up to six replaced teeth). In the multivariate analysis, the “opposite dentition” was also found to be a significant factor in the survival of the DCR-RDPs-SEFPs (*p* <.05). DCR-RDPs-SEFPs, where the opposite jaw was fitted with a telescopic cast prosthesis, showed a reduced risk of loss of function of about 56%. For full dentures and other partial dentures (including implant-supported dentures) in the opposite jaw, the risk of functional loss was 74% and about 85% lower, respectively (Table 4).

**Table 4:** Cox Regression model for DCR-RPDs with spark-eroded friction pins

Variables	B	*p*-value	Exp(B)	95% Confidence interval of Exp (B): Upper Lower	
Gender- female	0.448	0.112	1.565	0.900	2.722
Localization Mandibula	0.397	0.177	1.488	0.836	2.650
Opposing dentition		< 0.05 ^α^			
DCR-RPDs	−0.816	< 0.05 ^α^	0.442	0.221	0.884
Full dentures	−1.344	< 0.01 ^α^	0.261	0.096	0.712
Implant-supported RDPs	−1.869	< 0.05 ^α^	0.154	0.033	0.728
Kennedy-Class		< 0.01 ^α^			
Kennedy-Class II	1.139	< 0.01 ^α^	3.123	1.581	6.169
Kennedy-Class III	−0.644	0.329	0.525	0.144	1.916
Number of abutments		0.199			
3 to 4 abutments	−0.455	0.118	0.635	0.359	1.122
5 to 6 abutments	−0.685	0.517	0.504	0.064	3.993
7 and more abutments	1.287	0.241	3.623	0.421	31.170
Number of replaced teeth		< 0.001 ^α^			
7 to 10	1.386	< 0.05 ^α^	4.000	1.354	11.822
11 and more	2.889	< 0.001 ^α^	17.982	5.684	56.883

B=coefficient; Exp(B)=hazard; Reference for the hazard is the respective missing subgroup;^α^=significant influence

Of all the other variables tested, none showed a significant influence on the survival probability of the prostheses.

### Abutment teeth

Within the mean follow-up of 14.07 ± 7.89 years (max 31.66 years), 563 abutment teeth were observed, of which 39.43% (*n* = 222) had to be removed due to multiple complications (e.g. decay, loosening, inflammation, or fracture). Mean survival time for abutment teeth was 20.29 ± 0.51 years (95% CI: 19.29 to 21.29 years). The 5-, 10-, 15-, 20-, 25-, and 30-year survival times of all abutment teeth are displayed in Table 2.

The Kennedy Class classification showed a significant influence on the survival rate of the abutments (*p* <.05). The mean survival time for Kennedy Class I abutments was 19.90 ± 0.67 years (95% CL: 18.58 to 21.22 years), for Class II abutments was 17.50 ± 0.91 years (95% CL: 15.73 to 19.28 years), and for Class III abutments was 25.17 ± 1.10 years (95% CL: 23.03 to 27.32 years). The 5-, 10-, 15-, 20-, 25-, and 30-year survival times of all abutment teeth depending on Kennedy Class are displayed in Table 3. Kennedy Class II abutments had a 2.5-fold increased risk of removal compared to Kennedy Class I abutments in the multifactorial analysis (Table 5).

**Table 5:** Cox Regression model for abutment teeth

Variables	B	*p*-value	Exp(B)	95% Confidence interval of Exp (B): Upper Lower	
Gender- female	0.282	0.069	1.325	0.978	1.797
Localization Mandibula	0.492	< 0.01 ^α^	1.635	1.200	2.227
Opposing dentition		< 0.001 ^α^			
DCR-RPDs	−0.687	< 0.001 ^α^	0.503	0.348	0.727
Full dentures	−1.154	< 0.001 ^α^	0.316	0.187	0.532
Implant-supported RDPs	−0.958	< 0.05 ^α^	0.384	0.185	0.794
Kennedy-Class		< 0.001 ^α^			
Kennedy-Class II	0.907	< 0.001 ^α^	2.476	1.700	3.605
Kennedy-Class III	−0.166	0.567	0.847	0.479	1.496
Number of abutments		< 0.05 ^α^			
3 to 4 abutments	−0.301	0.090	0.740	0.522	1.048
5 to 6 abutments	−0.582	0.098	0.559	0.280	1.114
7 and more abutments	0.691	0.138	1.997	0.800	4.983
Number of replaced teeth		< 0.001 ^α^			
7 to 10	0.480	< 0.05 ^α^	1.616	1.020	2.558
11 and more	1.669	< 0.001 ^α^	5.304	3.222	8.733
Vitalitity		< 0.05 ^α^			
Root canal treated	0.133	0.610	1.142	0.685	1.904
Root canal treated + core build up	1.029	< 0.05 ^α^	2.799	1.239	6.323
Probing depth		< 0.001 ^α^			
4 to 5 mm	0.224	0.330	1.252	0.797	1.967
≥ 6 mm	0.942	< 0.001 ^α^	2.564	1.573	4.180
Crown-Root length ratio ≥ 1.8:1	0.404	< 0.05 ^α^	1.497	1.078	2.080

B=coefficient; Exp(B)=hazard; Reference for the hazard is the respective missing subgroup;^α^=significant influence

The variable number of teeth replaced also had a significant effect on the survival time of the abutment teeth (*p* <.05). The mean survival time of the abutments in group 1 was 24.33 ± 0.92 years (95% CL: 22.53 to 26.14 years), in group 2 22.27 ± 0.81 years (95% CL: 20.68 to 23.86 years), and in group 3 15.50 ± 0.72 years (95% CL: 14.09 to 16.92 years).

**Fig. 3:**
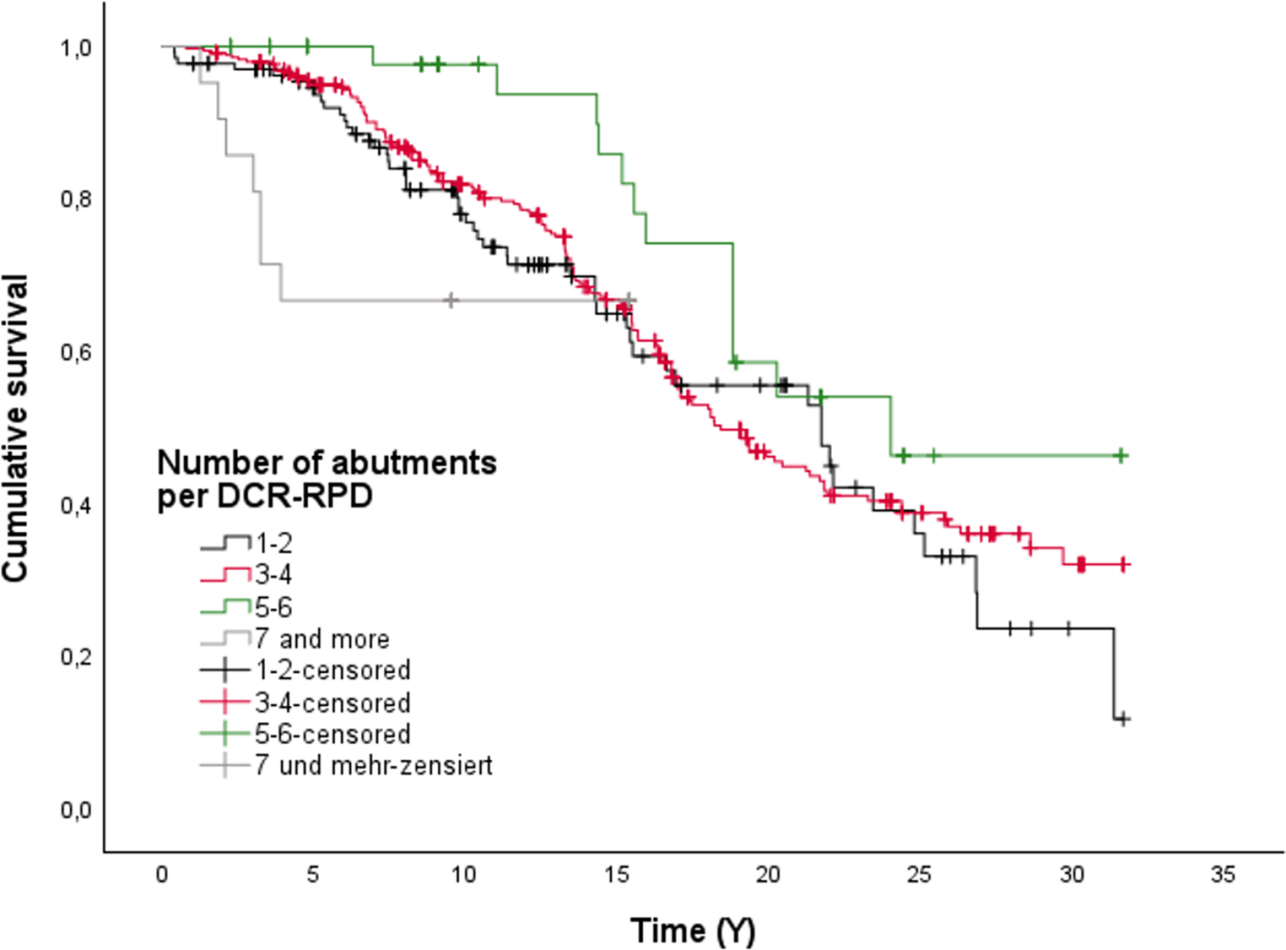
Outcome probability of all DCR-RPDs dependent on number of abutment teeth in each prosthesis (target event: tooth loss, n=563; Kaplan-Meier)

The number of abutment teeth in each prosthesis had a significant effect on the survival time of the individual abutment teeth (*p* <.05). The mean survival time of the abutments was 19.57 ± 1.09 years (95% CL: 17.43 to 21.70 years) in group 1, 20.29 ± 0.62 years (95% CL: 19.08 to 21.50 years) in group 2, 23.87 ± 1.56 years (95% CL: 20.81 to 26.93 years) in group 3, and 11.14 ± 1.31 years (95% CL: 8.56 to 13.71 years) in group 4 (Fig. 3).

In addition, the variables probing depth and crown-root length ratio significantly influenced the survival of the abutment teeth (*p* <.001). The mean survival time of abutment teeth that had pocket depths of less than 3 mm in at least one location or a maximum of 3 mm in at least two locations was 22.09 ± 1.06 years (95% CL: 20.02 to 24.17 years). For abutment teeth with pocket depths of 4–5 mm in at least three areas, the mean survival time was 21.22 ± 0.66 years (95% CL: 19.93 to 22.50 years). The mean survival time for abutment teeth with two pocket depths of 7 mm or more or at least two sites with a depth of at least 6 mm was 15.42 ± 1.04 years (95% CL: 13.38 to 17.46 years).

**Fig. 4:**
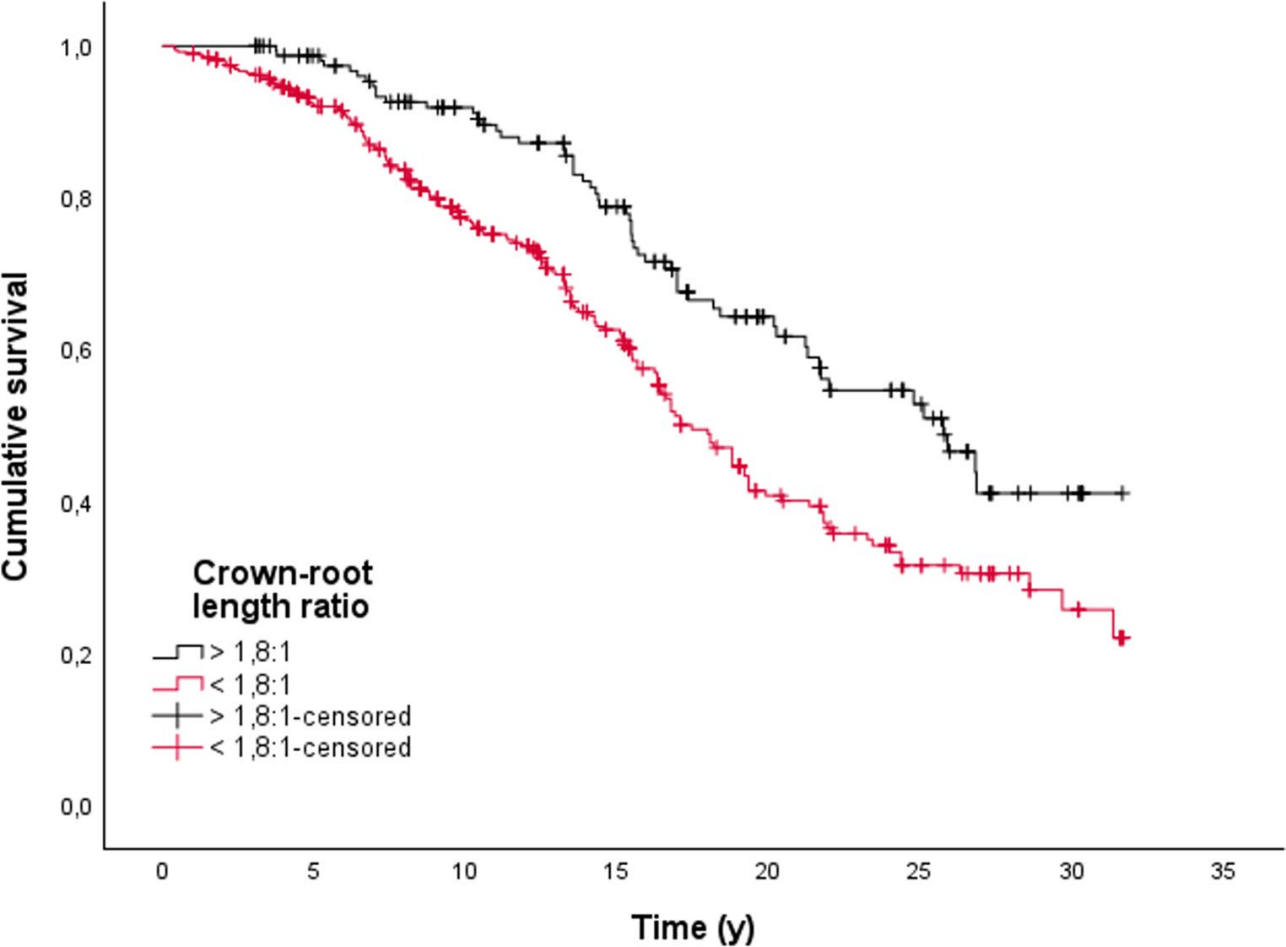
Outcome probability of all DCR-RPDs dependent on crown-root length ratio of the abutment teeth (target event: tooth loss, n=563; Kaplan-Meier)

The mean survival time of abutment teeth with a crown-root length ratio of less than 1.8:1 was 23.16 ± 0.83 years (95% CL: 21.52 to 24.79 years). For abutment teeth with a crown root length ratio of 1.8:1 or greater, the mean survival time was 18.93 ± 0.62 years (95% CL: 17.71 to 20.16 years). The survival rates of the abutment teeth related to the crown root length ratio are shown in Fig. 4.

In the multifactorial analysis, abutment teeth with probing depth group 3 had an approximately 2.6-fold increased risk of extraction compared with group 1. Furthermore, abutment teeth with a crown-to-root length ratio of 1.8:1 or greater were approximately 1.5 times more likely to be removed than abutment teeth with a length ratio below this value. Also, teeth with root canal fillings and post restorations had a 2.8-fold increased risk of extraction in comparison to vital teeth in the multifactorial analysis (Table 5).

Of all the other variables tested, none showed a significant influence on the survival probability of the abutment teeth.

### Aftercare measures

A total of 1418 follow-up procedures were performed during the observation period of up to 30 years (Table 6). At least one follow-up procedure was required for all DCR-RPDS-SEFPs examined, including their abutment teeth, during the observation period. The most common follow-up measures related to the prostheses were reline (*n* = 294), repair of veneering (*n* = 186), and repair of acrylic denture parts (*n* = 93). For the abutment teeth, the most common measures were reattachment of primary crowns (*n* = 340), root canal treatment (*n* = 156), and tooth filling (*n* = 109). The most common follow-up procedures were reattachment of primary crowns (*n* = 340), followed by relining of the denture base (*n* = 294) and repair of veneers (*n* = 186).

**Table 6:** Aftercare treatment

Aftercare measures	Number (*n*)	Percent (%)
Reattachment of primary crowns	340	24.0
Reline	294	20.7
Repair of facing	186	13.1
Root canal treatment of abutment tooth	156	11.0
Abutment tooth filling	109	7.7
Post core build-up	105	7.4
Repair of acrylic denture parts	93	6.6
Adding prosthetic tooth	89	6.3
Friction componenet activation	46	3.2
Total	1418	100

## Discussion

### Key results

Multiple of the tested variables showed a significant influence on the survival of either the prostheses or the abutment teeth. Kennedy class, the number of teeth replaced per prosthesis and the opposite dentition showed a significant influence on the survival probability of the DCR-RPDs-SEFPs. In contrast, the variables gender, number of abutments, and localization had no effect on their survival. Therefore, the null hypothesis could be rejected. Similarly, the null hypothesis concerning the abutment teeth could be rejected, as the following factors had a significant influence on their survival probability: Kennedy class, the number of teeth replaced per prosthesis, the number of abutment teeth per prosthesis, probing depth, crown-root length ratio and vitality. The variable gender, localization, and opposing dentition had no effect on the survival of the abutments.

### Limitations

The mean observation period was 14.07 ± 7.89 years in the present study, and the maximum was 31.66 years. Due to the long observation period of the present study and the small number of studies of similar length, comparability with the literature reviewed is limited. Moreover, there are only a few comparable studies that are dealing with DCR-RPDs with spark-eroded friction pins, and both are set at universities [1–3, 12, 13]. Studies with a similarly long observation period are rare. Apart from the studies by Yoshino et al. and Prott et al., no other studies have reached a maximum observation time that is as high [4, 5].

Apart from the methodological problems mentioned above, the study has many positive aspects. The prostheses were always manufactured in the same dental laboratory so that standardized restorations of comparable quality can be assumed. The initial treatment as well as the final fitting of the prostheses was always performed by the same practitioner, whereas follow-up measures were also carried out by other practitioners. Nevertheless, it should be noted that this could also be seen as a limitation of the study, given that the skills of both technician and dentist may have improved over the years, leading to variations in the quality of the dentures over time. Continuous and date-related data collection was carried out regarding the manufacturing process, the follow-up examinations, and the necessary post-treatment measures within the private dental practice, thereby ensuring high data quality. However, due to the free choice of dentist or a temporary or permanent change of dentist, 100% data collection cannot be guaranteed.

The statistical analysis considered potential influencing factors, with their effects on the survival of the DCR-RPDs-SEFPs and abutments being assessed using the Kaplan-Meier method and Cox regression. However, potential correlations and/or interactions between certain variables, as well as overlaps in specific aspects, could not be fully excluded, which represents a limitation of the study.

### Interpretation

The calculated 5-, 10-, 15-, 20-, 25-, and 30-year survival probabilities of 97.9%, 85.3% and 74.4%, 58.9%, 51.2% and 42.0% for DCR-RPDs-SEFPs are in accordance with the current literature [1–11, 19, 20]. The study by Hinz et al. observed 182 DCR-RDPs with spark-eroded friction pins in a dental hospital setting, and showed a lower overall survival probability of the prostheses after 10 years of 65.5% in comparison to the present study [1]. Contrary to this, Yoshino et al. reported 10 – and 20- year survival rates of 94.7% and 70.8% for DCR-RPDs [4].

In the present study, Kennedy Class II prostheses had a three times higher risk of loss of function compared to Class I prostheses, which can be explained by the unfavorable static leverage effects in Kennedy Class II prostheses. In contrast, Wöstmann et al., who also investigated the impact of the Kennedy class on the survival of DCR-RPDs, demonstrated that this was not a significant factor influencing their survival [9]. However, it should be noted that Kennedy Class I presented the largest group in the present study (*n* = 121), followed by Kennedy Class II (*n* = 50) and Kennedy Class III (*n* = 25). Kennedy Class IV is not present in this study. This is since DCR-RDPs are mainly fabricated for patients with few residual abutments and fixed dental prosthetics, and implant restorations are more suitable for a Kennedy Class IV. Due to this methodological fact, the present result must be viewed critically, and further research is needed to explore the implications of this finding.

This study also showed that the number of teeth replaced had a highly significant influence on the survival of DCR-RPDs-SEFPs (*p*<.001). With seven to ten teeth replaced and eleven or more teeth replaced, the risk of loss of function was 4 times and 18 times higher, respectively. This result can be explained by the fact that as the number of replaced teeth increases, the number of supporting abutment teeth decreases, thereby reducing stability and ultimately increasing the inherent mobility of the prosthesis. Similar findings are made by Hinz et al., who observed DCR-RDPs with spark-eroded friction pins and showed a significantly higher survival time of the prostheses in the group of non-severely reduced dentition (more than three abutment teeth) in comparison to the group of severely reduced dentition (three or less abutment teeth) [1, 2]. A study by Prott et al. also found that DCR-RPDs with 3 or fewer abutments had a lower probability of survival [5]. The study by Eisenburger et al. found that an increased number of abutment teeth significantly improved the longevity of prostheses and abutment teeth in most DCR-RPD designs, but this was not the case for bilateral free-end saddle designs without an anterior bounded saddle [10]. Multiple other studies have shown that the number of abutment teeth has a significant to highly significant influence on the survival probability of DCR-RPDs; therefore, restorations with fewer than three abutment teeth had a significantly poorer prognosis [3, 4, 6–9, 14–16]. Unlike many other studies, the present study found that the variable number of abutment teeth per prosthesis had no significant influence on DCR-RPDs-SEFPs survival (*p* >.05).

In accordance with the current literature, the 5-, 10-, 15-, 20-, 25- and 30-year survival probabilities of 94.1%, 81.4%, 67.5%, 48.4%, 38.8% and 31.4% for abutment teeth are comparable with the current literature [4, 5, 16, 19, 20, 22]. Prott et al. reported survival probabilities of 94.3%, 80.2%, 51.0% and 27.0% after 5, 10, 20 and 30 years, respectively, for abutment teeth [5].

Compared to Kennedy Class I, the abutment loss rate for Kennedy Class II was 2.5 times higher. Kennedy Class II involves a unilaterally shortened dental arch. This can result in unfavorable leverage because the teeth to be replaced are outside the support polygon.

In contrast to the present study’s investigation of factors influencing prosthesis survival, the number of abutment teeth per prosthesis was found to have a significant influence on abutment survival (*p* <.05). Hinz et al. also reported that abutment teeth in prostheses with three or fewer abutment teeth showed significantly lower survival probabilities than in prostheses with four or more abutments [12, 13]. This has been reported in other studies [4, 7–9, 16]. In contrast, the Cox model showed no significant effect. However, Dittmann et al. concluded that the number of abutment teeth had no significant effect on the probability of abutment tooth survival [14]. Wenz et al. also found that the risk of abutment loss did not increase significantly with ≤ 3 abutment teeth per prosthesis [3].

The number of teeth replaced also had a significant effect on abutment survival. The risk of extraction was 1.6 times higher for “7 to 10 teeth replaced” and 5.3 times higher for “11 and more” teeth replaced compared to “up to less than 6” teeth replaced. Replacing more teeth means there are fewer abutment teeth, and each abutment tooth has to take on more load. If there are more abutment teeth or fewer teeth to replace, the force can be better distributed and overloading of the abutment teeth avoided.

The present study showed that the probability of survival of abutment teeth decreases steadily with increasing pocket depth. The Cox model showed that abutment teeth with pocket depths of 7 mm or more, or at least 6 mm in two or more places, had an approximately 2.6-fold increased risk of extraction. This should be considered when planning DCR-RPDs, especially in cases with severely reduced dentition (≥ 3 abutment teeth) [18, 20].

Furthermore, it was observed that abutment teeth with a crown-to-root length ratio of 1.8:1 or greater had an approximately 1.5 times higher risk of extraction compared to abutment teeth with a length ratio below the above-mentioned ratio. Yoshino et al. reported a 2.28 higher risk of failure for abutment teeth with a shorter inner crown [4].

The vitality of the abutment teeth showed a highly significant influence on the survival of the abutment teeth (*p*<.001). Nonvital abutment teeth with root canal fillings and post restorations had a 2.8-fold increased risk of extraction in comparison to vital teeth in the present study. This is also confirmed by other studies [5, 12–14, 21, 22]. The study of Oh et al. reported that the risk of abutment loss was approximately three times greater with the loss of vitality [21]. This was also demonstrated in a study by Vogler et al., which reported significantly poorer survival rates for abutment teeth with posts and core build-ups in DCR-RPDs in comparison to vital abutment teeth [22]. Hinz et al. reported higher fracture rates after 120 months in endodontically treated teeth, but no difference in performance between teeth with post and core reconstructions and root fillings only [12].

In this study, the reattachment of primary crowns (24%), the relining of dentures (20.7%), and the repair of veneers (13.1%) were the three most common follow-up procedures. The number of total aftercare measures as well as the most common aftercare measures are in comparison with the current literature [1, 9, 11, 16, 17, 20]. Schwindling et al. observed 117 DCR-RPDs and 385 abutment teeth over seven years and reported debonding of primary crowns (34%), fracture of the denture base (17%), relining (12%) and failure of the veneer of the secondary crown as the most common aftercare measures [17]. Verma et al. reported that gingival inflammation, periodontal disease, caries, debonding of primary crowns and loss of facings were the most common complications [20]. Wöstmann et al. observed parallel-sided double-crowns over 5 years and reported relines (34.8%), facing repairs (26.9%), and debonding of primary crowns (20.6%) as the most common aftercare measures, while friction loss occurred in 3% only [9]. In the current observation, a total of 46 spark-eroded friction pins were reactivated, accounting for 8.1% of all abutment teeth. Considering the long observation period of up to 30 years, this demonstrates that spark-eroded friction pins are an effective way for restoring friction. Moreover, this process does not require laboratory time and can be performed chairside.

## Conclusion

In this study, DCR-RPDs-SEFPs showed good results in terms of longevity during a 30-year maximum observation period. The study was conducted in a private dental practice, demonstrating the procedure’s ease of performance outside a university setting. Nevertheless, the following should be considered when planning DCR-RPDs-SEFPs: Kennedy Class III DCR-RPDs-SEFPs demonstrated the highest survival rates, while prostheses replacing 11 or more teeth exhibited the lowest survival rates. In terms of the longevity of the abutment teeth, the number of abutment teeth, pocket depth, vitality and crown/root length ratio should also be considered.

## Data Availability

No datasets were generated or analysed during the current study.
